# Re: The medical evidence on non-therapeutic circumcision of infants and boys—setting the record straight

**DOI:** 10.1038/s41443-022-00579-z

**Published:** 2022-07-05

**Authors:** Brian J. Morris, Stephen Moreton, John N. Krieger, Jeffrey D. Klausner, Guy Cox

**Affiliations:** 1 School of Medical Sciences, University of Sydney, Sydney, NSW 2006 Australia; 2 CircFacts, 33 Marina Avenue, Warrington, WA5 1HY UK; 3 Department of Urology, University of Washington School of Medicine, Seattle, WA 98194 USA; 4 Department of Medicine, Population and Public Health Sciences, Keck School of Medicine of the University of Southern California, Los Angeles, CA 90033 USA; 5 Australian Centre for Microscopy and Microanalysis, University of Sydney, Sydney, NSW 2006 Australia

**Keywords:** Risk factors, Preventive medicine

We read with interest a review by Deacon and Muir (‘D&M’) that concluded so-called ‘non-therapeutic’ male circumcision (NTMC) of infants and children provides insufficient benefits and that risks were too high for it to be recommended in the UK [1]. Instead, they suggest delay until the boy is old enough to make his own decision. However, in contrast to the UK, policy statements by the American Academy of Pediatrics (AAP) [2] and Centers for Disease Control and Prevention (CDC) [3], finding benefits of NTMC exceed risks, are evidence-based.

Flaws in D&M’s arguments include reliance on small, weak, out-of-date or inappropriate studies contradicted by more recent high-quality evidence. Unlike systematic reviews and meta-analyses, D&M did not engage sufficiently with existing evidence. Studies cited were not rated by quality.

They ignored a landmark high-quality study by CDC researchers of adverse events from 1.4 million neonatal and older age US males [4]. NTMC risk in infants was 0.4% and was 20-fold higher at age 1–9 and 10-fold higher at age ≥10. Similar values were cited in the AAP’s policy statement. Thus D&M’s claim of 1–5% risk may apply in non-US countries or to later circumcision.

Also missing were key studies and important critiques of various publications they cited, as well as meta-analyses, and systematic reviews of benefits and risks (summarised in ref. [5]). If included, D&M’s overall conclusion would have been more balanced. Instead of number-needed-to-treat (NNT) for each condition, they should have combined all such information into an overall risk-benefit analysis. An informed ‘big picture’ might then have emerged to better inform parents and practitioners. Several risk-benefit analyses have been published over the past decade, including ours in ‘Mayo Clinic Proceedings’ cited by the CDC. The only one for the UK found benefits exceeded risks by 200:1, with failure to perform NTMC in infancy likely resulting in at least one adverse medical condition among over half of uncircumcised males during their lifetime [6]. Contrary to D&M’s assertion, the data we used for risk-benefit analyses were not ‘over-estimates,’ but came from high-quality studies.

Contrary to their claim that the foreskin becomes fully retractile in 99% by age sixteen, our systematic review, involving 43 studies, included one finding full retractability was 51.1% in 1834 uncircumcised adolescent boys [7] and averaged 96.6% in men, being 92.2% in British National Servicemen [8].

D&M criticise a meta-analysis of lifetime UTI risk (D&M-ref22) because it contained only one study of men. But men in that study attended a STI clinic with infection symptoms, whereas the two studies D&M suggested for inclusion lacked UTI cases. D&M ignored the meta-analysis group aged 1–16 years. Thus, D&M’s NNT > 100 claim is misguided as it applies to infants only. Eisenberg et al. found number of NTMCs needed to prevent one UTI in infants was 39, decreasing to 29 when other sequelae were included [9]. In comparison, childhood vaccination prevents one outpatient visit and one hospitalisation for influenza for every 50 and 1031–3050 vaccinated children, respectively [10].

D&M argue that infant NTMC would result in more boys needing antibiotics for postoperative wound infection than would need them for a UTI. Their claim is based on a 10% estimated post-NTMC wound infection prevalence. But this value contradicted their earlier statement that the overall complication rate for infant and paediatric NTMC is 1–5%. How can the prevalence of one specific complication (infection) be higher than the overall prevalence?

To resolve this, we translated the German language narrative review they cited for the 10% figure (D&M-ref125) and followed the reference trail through two further review articles to the primary source: [11]. This cited two very small *n* values for infections: *n* = 2 (4%) for boys circumcised with a Plastibell device and *n* = 5 (10%) for boys circumcised with scissors. Thus, the 10% figure is a maximum value in a small study, applies to an old method, and is for older boys, not infants, so is misleading. D&M also assume that all of those 10% would require antibiotics. In fact, most such infections are superficial and resolve with local treatment. A study of 5521 NTMCs noted infection in 23 (0.4%) [12]. Of these, only 4 (17%) required antibiotics, the rest resolving with topical antiseptics.

In contrast, antibiotics are advised for all UTIs in infants, even if the UTI is merely suspected. Based on a NNT of 100 for infant UTI prevention by NTMC and the estimate of 0.4% for infections [12], instead of the much lower 0.0834% in the large US study [4] (where only 2.2% of those, i.e., 0.0018%, were likely NTMC-related), one can calculate *n* = 10 UTIs prevented from 1000 circumcisions, and *n* = 4 wound infections. If the figure of 17% of wound infections requiring antibiotics is representative, then 0.7 of those 4 wound infections would need antibiotic treatment, as opposed to all ten UTIs. Infant NTMC therefore results in a substantial net reduction in antibiotic use even when erring in D&M’s favour, and without considering later UTIs and other infections also prevented by infant NTMC. When coupled with the increasing problem of antibiotic resistance, particularly in relation to infant UTIs, NTMC in infancy represents a significant benefit.

For STIs, D&M fail to explain how in socioeconomically advantaged countries most HIV infections occur from receptive anal intercourse in men-who-have-sex-with-men (MSM). In insertive MSM, risk is substantially lower in those circumcised. D&M suggest that some men forego condom use after circumcision, but ignore a 2021 meta-analysis by Gao showing no such decline in condom use. While current HIV treatments extend lifespan, D&M did not acknowledge patients’ lifelong elevated risk of HIV-associated comorbidities. D&M refer to studies by Van Howe, but not the numerous critiques of his methods and flawed statistics [5]. Castellsagué et al.’s critique was titled: ‘HPV and circumcision: A biased, inaccurate and misleading meta-analysis.’

Their review of penile cancer was misleading. In all studies, penile cancer prevalence was much lower in circumcised men. NNT for uncircumcised males was 900 for Denmark and 600 for the US [13]. Based on average UK male life expectancy of ~79 years, 33.15 million male population, and ~700 cases/annum (Cancer Research UK), one can calculate a NNT of ~600 for the UK if penile cancer were unique to uncircumcised males, and ~1000 if only three-times higher. Circumcised men are also at lower risk of prostate cancer. Increasing circumcision prevalence in the UK from the current ~20% to ~90% should result in fewer cases.

D&M misconstrue a multinational study by Castellsagué et al. of HPV and cervical cancer (D&M-ref68) which included not just male partners of high-risk, but also those of intermediate risk. Contrary to D&M, RCT data exist. While HPV vaccination has lowered HPV prevalence in the UK, only 64.9% of year 9 females completed the 2-dose course in year 2019/2020, quadrivalent and nonavalent HPV vaccines cover 2 low-risk types, and 2 and 7, not all 14, oncogenic HPV types, and lifelong effectiveness is not assured.

Our recent meta-analysis of all 27 studies (1.5 million males), that included D&M’s reference 85, found risk of meatal stenosis was 0.656% [14]. Most diagnoses are actually a ventral ‘meatal web’ and are asymptomatic, so clinically nonsignificant.

Because adverse event risks are 10–20-fold higher for circumcision of non-neonatal males [4], and circumcision reduces risk of infections and other conditions over the lifetime, NTMC is cost-saving.

For pain, effective local anaesthetic methods are mandated. The CDC stated, ‘painless circumcision [by Gomco clamp] is possible in almost all [93.3%] newborns if it is performed during the first week of life.’ D&M’s reference 83 disputed those figures and other data finding <2% experience excessive pain. D&M miscommunicate a survey of parents’ perceptions during the 6 weeks following their newborn sons’ circumcision (D&M-ref103). Pain scores were not increased ‘for up to 6 weeks,’ and the study stated, ‘no long-term adverse effects were noted in the 6 weeks of follow-up.’ D&M claim that NTMC pain has long-term effects but cite as support pain during neonatal intensive care management, heel sticks and cardiac surgery. Contradicting speculation that NTMC pain causes central nervous system changes affecting empathy, a 2020 study by Miani found no such association.

Sexual function was addressed, but rather than a balanced overview of the considerable physiological and epidemiological evidence, as well as RCTs and meta-analyses finding similar or better function in circumcised men, D&M cite seriously flawed studies (D&M-refs114&123); see [5] for critiques. In addition, D&M cite an online post by Van Howe (D&M-ref113) of his peer-review of the CDC’s draft policy. This failed to sway the CDC.

Table 1 summarises the advantages of infant NTMC as compared to later age circumcision. Our extensive systematic review of the contrasting evidence found that high-quality data supports NTMC [5]. NTMC is, moreover, legal and ethical. Jacobs 2013 interpreted Article 24(3) of the United Nations Convention on the Rights of the Child [15] as favouring NTMC, since not circumcising boys has been deemed as prejudicial to their health. The AAP and CDC found benefits of NTMC exceeded risks, and, noting cultural sensitivities, recommended parental choice, education, insurance coverage, and provider training.

**Table 1:** Issues to consider for timing of male circumcision: neonatal vs. later.

Neonatal circumcision	Circumcision of older boys and men
• Simple.	• More complex.
• Quick (takes several minutes).	• Half an hour or more to perform.
• Cost is lower.	• Much more expensive (often unaffordable).
• Low risk (adverse events 0.4%).	• Moderate risk (adverse events 4–8%).
• Bleeding (uncommon) is minimal and easily stopped.	• Bleeding more common, requiring cautery or other interventions.
• Sutures not needed.	• Sutures or tissue glue needed.
• Convenient for patient.	• Inconvenient (time off school or work).
• Local anaesthesia for age <2 months.	• General anaesthesia for age >2 months to age 9 years. Local anaesthesia for men, although general anaesthesia often preferred by surgeon.
• Healing is fast (<2 weeks).	• Healing takes 6 weeks or more.
• Cosmetic outcome usually good.	• If stitches used stitch marks may be seen.
• No prior anxiety.	• Fear of undergoing an operation.
• Does not disrupt feeding or other day-to-day activities.	• Abstinence from sexual intercourse for the 6-week healing period.
• No embarrassment.	• May be embarrassed.
• Benefits start immediately after healing is complete.	• Benefits delayed. Meantime may suffer from medical problems that he would have been protected against if circumcised earlier.
• Avoids costs for treatment of later medical conditions that circumcision protects against.	• Cost of treatment of these, including both direct and indirect costs.

## Data Availability

For data referred to in this Comment article please email the first author.
